# A Case of Spontaneously Resolving Valsalva Retinopathy in a 29-Year-Old Patient

**DOI:** 10.7759/cureus.82906

**Published:** 2025-04-24

**Authors:** Dawid Woszczek, Aleksandra Górska, Sebastian Sirek, Dorota Wyględowska-Promieńska

**Affiliations:** 1 Students’ Scientific Society, Department of Ophthalmology, Faculty of Medical Sciences in Katowice, Medical University of Silesia, Katowice, POL; 2 Department of Ophthalmology, Professor Kornel Gibiński University Hospital Center, Medical University of Silesia, Katowice, POL; 3 Department of Ophthalmology, Faculty of Medical Sciences in Katowice, Medical University of Silesia, Katowice, POL

**Keywords:** increased venous pressure, internal limiting membrane, minimally invasive treatment, preretinal hemorrhage, valsalva retinopathy

## Abstract

Valsalva retinopathy (VR) occurs when a sudden rise in pressure within the thoracic or abdominal cavity results in a pre-retinal hemorrhage. The elevated venous pressure leads to increased blood flow through the retinal vessels, causing the rupture of peripapillary capillaries. This rupture leads to unilateral or bilateral pre-retinal hemorrhage, with blood pooling beneath the inner limiting membrane (ILM).

A 29-year-old patient presented to the Emergency Eye Care Unit of the Professor Kornel Gibiński University Hospital Center of the Silesian Medical University in Katowice due to deterioration of vision in the right eye over the past five days. On physical examination, the visual acuity (VA) in the right eye was 0.4, and the ocular pressure was 24 mmHg. In the left eye, the VA was 1.0, and the ocular pressure was 22 mmHg. A fundus examination showed a tear-shaped hemorrhage in the fovea. On admission, an optical coherence tomography (OCT) scan of the right eye was performed, confirming the presence of a hemorrhage under the ILM, measuring 524 μm × 246 μm. One month after the initial visit, the VA in the right eye improved to 1.0. The dimension of the hemorrhage, measured by OCT, had decreased to 153 μm × 62 μm. Effective management of VR includes close observation and the avoidance of strenuous physical activity, which gives the patient a chance to regain useful VA and avoid surgical treatment.

## Introduction

Valsalva retinopathy (VR) is a pre-retinal hemorrhage resulting from a sudden increase in pressure within the thoracic or abdominal cavity. First described by Duane in 1972 [1], this phenomenon is considered rare, with its exact epidemiology still not well-documented in the scientific literature. However, it is known to primarily affect individuals who engage in activities that provoke rapid and significant pressure changes, such as intense physical exertion, coughing, vomiting, labor, straining during defecation, or thorax-abdominal trauma [2-5].

The pathophysiology of VR is based on a sudden rise in venous pressure, which causes a corresponding increase in blood flow through the retinal vessels. This increased flow exerts pressure on the fragile peripapillary capillaries, leading to their rupture and the subsequent leakage of blood into the pre-retinal space. The hemorrhaged blood then accumulates beneath the inner limiting membrane (ILM) of the retina, which typically results in the characteristic appearance of a flame-shaped hemorrhage [6]. Such hemorrhages are often located near the optic disc, and their size and location vary depending on the severity of the event. The clinical presentation of VR usually includes a sudden, painless loss of vision in one or both eyes, often described as visual disturbances or the presence of floaters. Patients may also experience a temporary or permanent decrease in visual acuity (VA), particularly if the hemorrhage is large or involves the macular region [7-9].

While the condition is often self-limiting, with many patients experiencing spontaneous resolution of the hemorrhage, the severity and duration of visual symptoms can vary. Although the majority of VR cases resolve without intervention, treatment options - including laser photocoagulation, intravitreal anti-vascular endothelial growth factor (anti-VEGF) injections, or even vitrectomy - may be considered for more severe or persistent cases. These approaches aim to reduce the risk of further retinal damage and improve visual outcomes, especially in patients with significant symptoms or recurring hemorrhages [10].

## Case presentation

**Figure 1:**
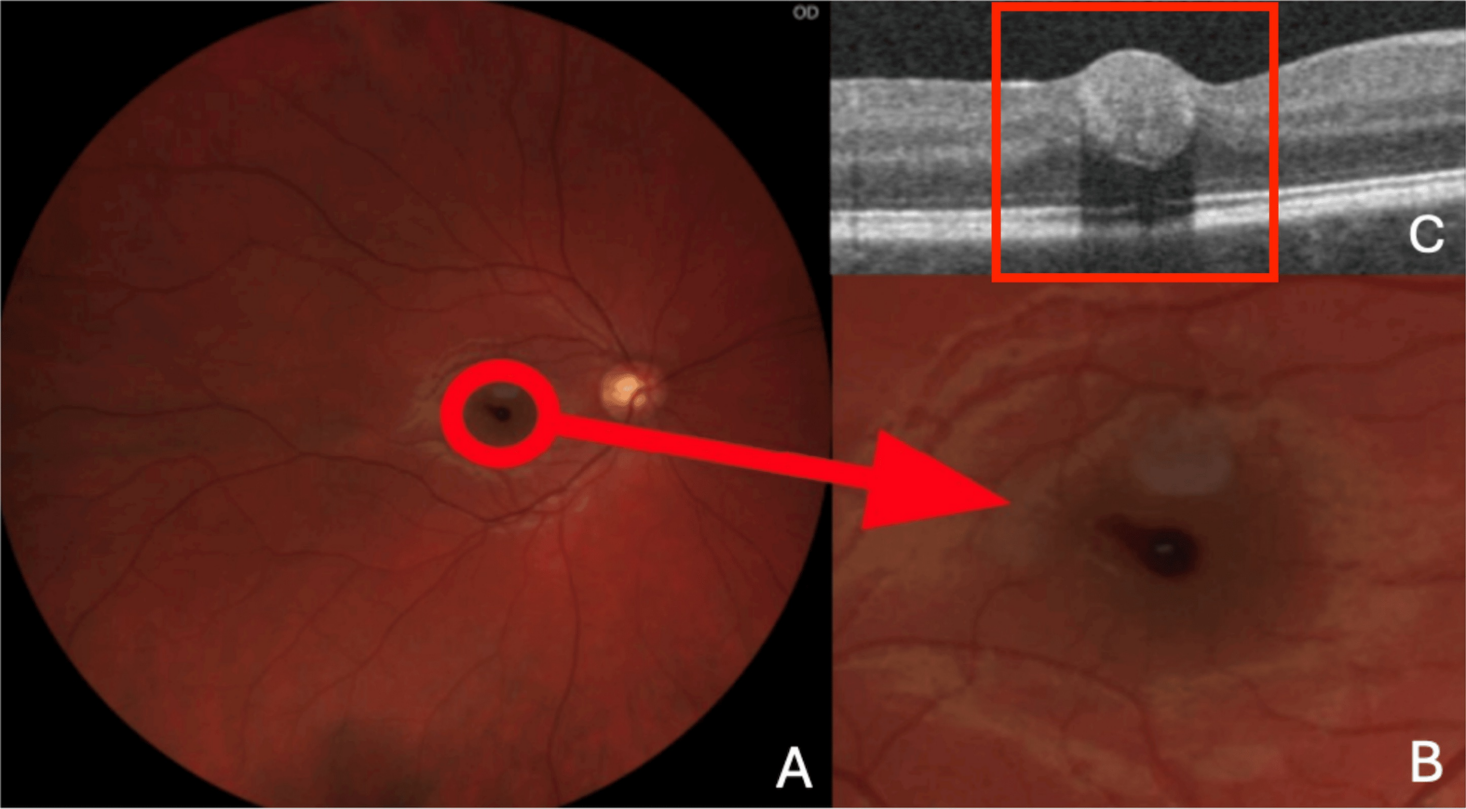
Fundus of the right eye, on the day the patient reported to the emergency room, and the corresponding OCT image. (A) Photograph of the fundus of the right eye, with a tear-shaped hemorrhage (arrow) in the fovea visible; (B) Close-up image of a tear-shaped hemorrhage (arrow) in the fovea; (C) OCT scan, with a tear-shaped hemorrhage (box) in the fovea visible. OCT, optical coherence tomography

A 29-year-old patient presented to the Emergency Eye Care of the Professor Kornel Gibiński University Hospital Center of the Silesian Medical University in Katowice due to deterioration of vision in the right eye for five days. In addition to the decreased VA, the patient reported in his history that he had been seeing a central spot, which had not moved with the eyeball’s movement, in the right eye for five days. The patient noticed the first signs of vision deterioration after bending down. In his history, the patient denied any comorbidities. On physical examination, VA in the right eye was 0.4, and intraocular pressure (IOP) was 24 mmHg; in the left eye, it was 1.0 mmHg and 22 mmHg, respectively. As shown in Figures 1A-1B, a fundus examination revealed a tear-shaped hemorrhage in the fovea, the size of which did not exceed that of one optic nerve disc.

**Figure 2:**
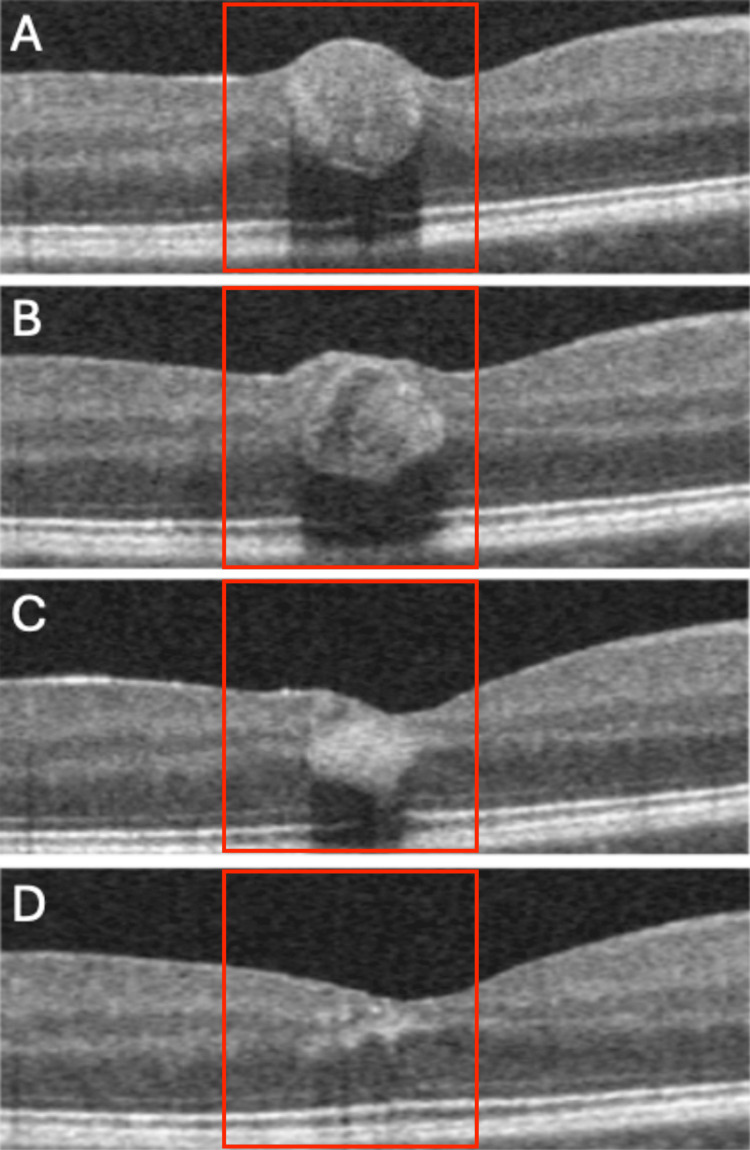
Subsequent follow-up of self-limiting intraretinal hemorrhage captured on OCT scans. (A) OCT image of hemorrhage on admission day; (B) OCT image after 1 week; (C) OCT image after 2 weeks; (D) OCT image after 4 weeks. OCT, optical coherence tomography

On admission, optical coherence tomography (OCT) (Optovue AngioVue; Optovue, Inc., Fremont, CA, USA) of the right eye was performed, which confirmed the presence of hemorrhage under the ILM, with dimensions of 524 μm × 246 μm. Observation and avoidance of physical activity were recommended. At the first follow-up, seven days after the patient's report, the VA of the right eye was 0.5 on examination. OCT of the right eye was performed again, and the size of the hemorrhage was measured, obtaining dimensions of 516 μm × 211 μm. The dimensions had decreased from the previous visit. In the subsequent follow-ups, which took place 14 days and one month after enrollment, respectively, the VA of the right eye was 0.6 and 1.0. On OCT, the dimensions of the hemorrhage decreased to 400 μm × 151 μm and 153 μm × 62 μm, respectively. All the above data have been presented in Figure 2 and Table 1.

**Table 1:** Subsequent follow-up of self-limiting intraretinal hemorrhage with dimensions and visual acuity.

Dimensions (µm)	Test date	Visual acuity
524 x 246	September 18, 2024	0.4
516 x 211	September 26, 2024	0.5
400 x 151	October 3, 2024	0.6
152 x 62	October 17, 2024	1.0

**Figure 3:**
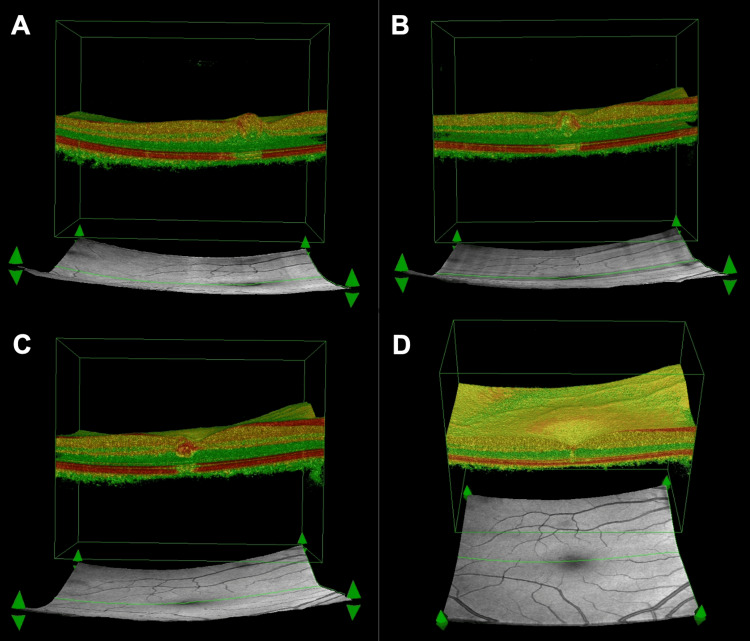
Process of spontaneous restriction of intraretinal hemorrhage in 3D OCT projection. Subsequent follow-up of self-limiting intraretinal hemorrhage with 3D OCT images with dates. (A) Visible intraretinal hemorrhage (date of the scan: September 18, 2024); (B) Visibly reduced intraretinal hemorrhage (date of the scan: September 26, 2024); (C) Visibly more reduced intraretinal hemorrhage (date of the scan: October 3, 2024); (D) Invisible intraretinal hemorrhage (date of the scan: October 17, 2024).

The process of spontaneous reduction of preretinal hemorrhage was visualized in the 3D OCT projection, as shown in Figure 3.

**Figure 4:**
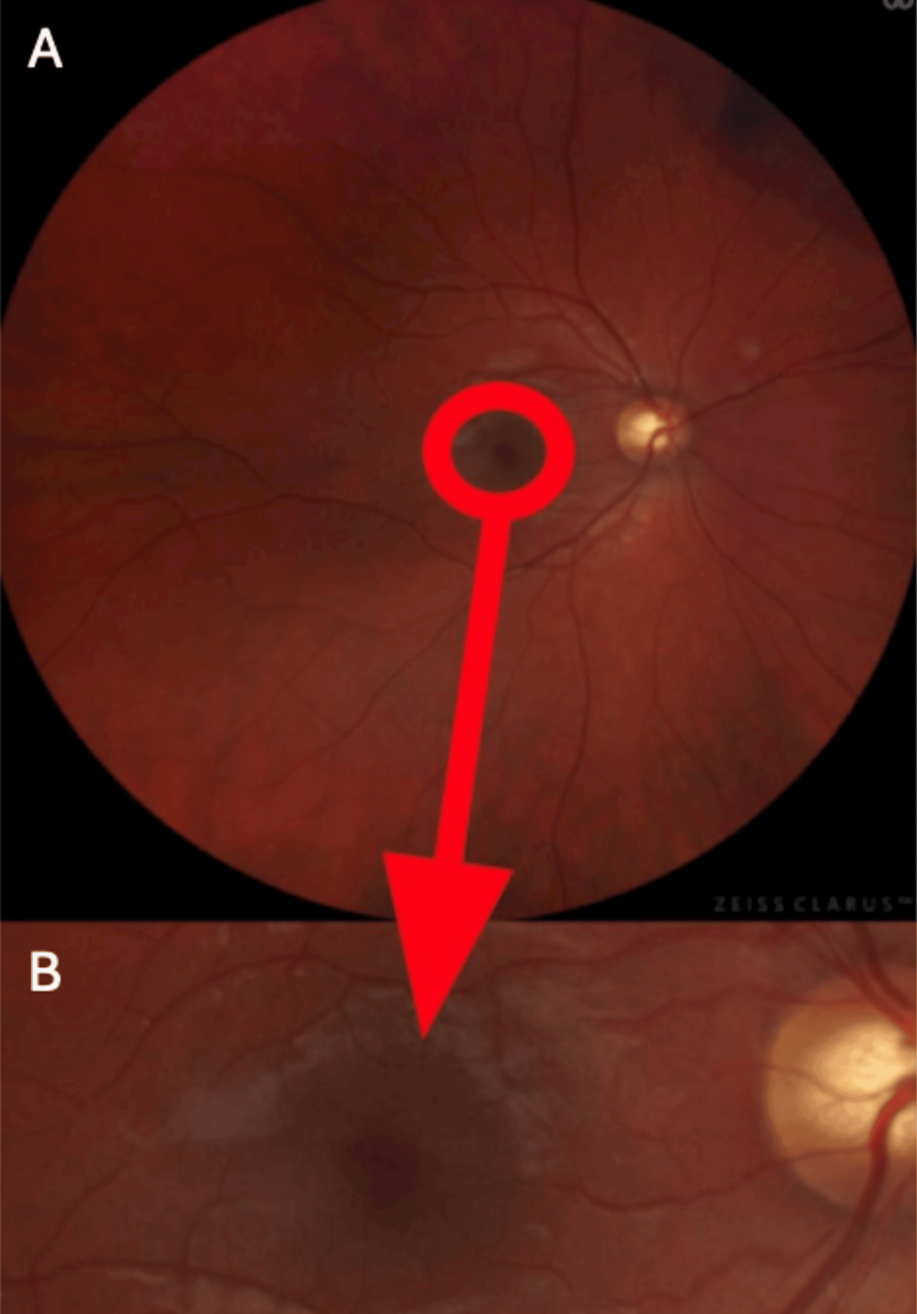
A fundus image showing the complete absorption (arrow) of the intraretinal hemorrhage. (A) Photograph of the fundus of the right eye showing the complete absorption of the hemorrhage; (B) Close-up photograph of the fundus of the right eye showing the complete absorption of the hemorrhage.

During subsequent follow-ups, self-limitation of the hemorrhage was observed until it completely disappeared after 35 days, achieving full return of VA and stabilization of IOP at 20 mmHg. The effect of restriction of the hemorrhage was captured on a fundus image, presented in Figure 4.

## Discussion

In the available literature, various treatment methods are considered, among the main options are observation, Nd-YAG (neodymium-doped yttrium aluminum garnet) laser hyaloidotomy, pneumatic displacement of hemorrhage by intravitreal injection of gas with or without recombinant tissue plasminogen activator (rTPA), and pars plana vitrectomy (PPV).

Mukherjee et al. highlight the fact that preretinal hemorrhage and sub-ILM hemorrhage resolve spontaneously in most cases, with a good visual outcome. Intervention with Nd:YAG laser or surgery is not necessary unless the patient wants a faster visual recovery, or there is a breakthrough hemorrhage into the vitreous. Gaseous injection of rTPA into the vitreous may be an effective treatment when the hemorrhage is in the deeper layers of the retina [11]. 

Studies by subsequent authors have shown that PPV is the most effective treatment for patients with severe intraretinal hemorrhage located under the ILM and insufficient spontaneous reabsorption. The most important intra- and post-operative complications of PPV use include secondary macular hole and accelerated cataract formation [12,13]. Hemorrhages with a diameter of less than one normal optic disc, as in the case described here, tend to resolve spontaneously in a short period of time, and a conservative approach is generally justified. In contrast, spontaneous resolution of large and dense hemorrhages is highly unlikely [13].

One common intervention is drainage with the Nd-YAG laser. The laser can be useful in treating preretinal hemorrhages that are not dense, have not coagulated, and when the blood is not localized under the ILM. Close proximity to the retinal surface risks the formation of a macular hole or a persistent pre-retinal defect. Further complications described include retinal detachment or epiretinal membrane formation [14,15].

Each time, the possibility of mislocalization of blood must be taken into account, even with advanced retinal imaging techniques, such as high-resolution OCT.

## Conclusions

Successful management of VR depends on the timing and size of the hemorrhage. Despite severe visual impairment in the early stages of the disease, VA is usually restored to normal levels. For hemorrhages smaller than the size of one normal optic disc, management includes close observation and avoidance of strenuous physical activity. This is a minimally invasive and safe method, especially for a young, active patient.
